# Surgeon-Applied Stress and a Ligament Tensor Instrument Provide a Similar Assessment of Preresection Flexion Laxity During Robotic Total Knee Arthroplasty

**DOI:** 10.1016/j.artd.2024.101450

**Published:** 2024-07-03

**Authors:** Catelyn A. Woelfle, Travis R. Weiner, Peter K. Sculco, Nana O. Sarpong, Roshan P. Shah, H. John Cooper

**Affiliations:** a Department of Orthopedic Surgery, New York Presbyterian Hospital - Columbia University Irving Medical Center, New York, NY, USA; b Department of Orthopedic Surgery, Hospital for Special Surgery, New York, NY, USA

**Keywords:** Total knee arthroplasty (TKA), Robotic-assisted total knee arthroplasty (RA-TKA), Gap balancing, Flexion laxity

## Abstract

**Background:**

Robotic-assisted total knee arthroplasty (RA-TKA) allows surgeons to perform intraoperative soft tissue laxity assessments prior to bone resections and is used to alter resections to achieve gap balance. This study compared 2 techniques for flexion gap laxity assessment during RA-TKA.

**Methods:**

A prospective study of 50 primary RA-TKAs performed by a single surgeon was conducted between February and October 2023. Following full exposure, anterior tibial dislocation, and osteophyte removal, maximal medial and lateral compartment flexion laxity was quantified to the nearest 0.5 mm by the robotic system using a dynamic, surgeon-applied stress (SURGEON). This data was used to plan a balanced flexion gap by adjusting the femoral component size, rotation, and anterior-posterior translation. Flexion laxity was quantified again after distal femoral and proximal tibial resections using a ligament tensor instrument (TENSOR). These new data were used to plan for the same desired flexion gap using the same variables. Paired-samples *t*-tests and a simple linear regression were used for analysis.

**Results:**

Both methods produced near-identical recommendations for femoral component sizing (mean deviation 0.06 sizes, range −1 to +1 size; *P* = .569), rotation (deviation mean 1.0°, range −3.0° to +3.0°; *P =* .741), and anterior-posterior translation (deviation mean 0.13 mm, range −0.5 to +0.5 mm, *P* = .785). SURGEON femoral component rotation predicted TENSOR rotation (R^2^ = 0.157; 95% confidence interval = 0.124, 0.633; *P* = .004).

**Conclusions:**

Assessing flexion laxity with a surgeon-applied stress vs a ligament tensor produced near-identical laxity data in RA-TKA, suggesting surgeons may comfortably choose either technique as a reliable method.

**Level of Evidence:**

Level III.

## Introduction

Robotic-assisted total knee arthroplasty (RA-TKA) has been shown to be more accurate and precise than conventional total knee arthroplasty (TKA) in alignment, component sizing, and component positioning [[1], [2], [3], [4], [5]]. In addition to precise bone resections, modern robotic systems also allow for intraoperative measurements of soft tissue laxity (eg, compartment gaps) in the medial and lateral compartments through a range of motion (ROM). Perhaps related to this ability to accurately assess soft tissue laxity, improvements in flexion and extension gap balance have also been demonstrated with the use of robotic guidance, with a recent study showing balanced flexion and extension gaps in 94% of RA-TKA compared to only 80% in conventional TKA performed with a measured resection technique [6].

Gap-balancing has become a popular technique in TKA. Understanding how tight or loose each compartment is prior to bone resections affords the opportunity to modify planned resection angles and resection depths prior to bone resections, with the goal of achieving balanced postresection gaps. This technique of planning slight modifications to bone resections in response to an individual patient’s soft tissue envelope, instead of always aiming for a neutral mechanical alignment, can be thought of as a personalized alignment strategy. For this concept of balancing through adjusted bone resections to be effective, the soft tissue laxity data must be accurate and reproducible. To help balance gaps through modifying bone resections, most RA-TKA systems allow surgeons to perform a pre-resection assessment of soft tissue laxity in medial and lateral compartments in both flexion and extension. The robotic platform can measure laxity in each compartment prior to any bony resections being made, based on the applied stress in extension and flexion.

Assessment of the extension gap laxity is relatively straightforward, as knee surgeons are familiar with and experienced in applying valgus and varus forces to the knee in extension to stress the medial and lateral sides, respectively. However, assessment of flexion gap laxity is less straightforward and more controversial, as there are several intraoperative techniques that can be used to assess flexion laxity. One common strategy is to measure flexion gap laxity based on manual surgeon-applied forces, which can be applied either through distraction or rotation. Some surgeons apply these forces manually with their hands, while others use levers such as a retractor or elevator to manually deliver distraction force. Another strategy is to utilize a specially designed ligament tensor instrument to distract the flexion space independently in both medial and lateral compartments [7], of which several different instruments exist.

The current study was designed to evaluate for any potential differences between 2 of these gap balancing techniques during assessment of soft tissue laxity in flexion. We investigated any differences in planned variables affecting the flexion gap, namely femoral component size, femoral component rotation, and the anterior-posterior (AP) position of the femoral component. Our hypothesis was that there would be no significant differences between these 2 commonly employed assessment techniques.

## Material and methods

This study was designed as a review of prospectively collected intraoperative data from 50 consecutive primary TKA cases from February 2023 to October 2023, in which these variables were collected and performed by a single fellowship-trained arthroplasty surgeon with more than 5 years of experience using robotic TKA. This study was approved by our institution’s international review board (IRB-AAAU9786).

A total of 50 RA-TKA cases were included. The primary outcome variable used to justify sample size was femoral component rotation. A power analysis for comparing paired differences revealed a necessary sample size of at least 34 patients to determine a mean difference of ≥1 degree in a paired samples *t*-test with a standard deviation of 2 degrees, 80% power, and *P* < .05.

Within the sample of 50 cases, the mean age of the patients included in the study was 68.5 years (range, 55-88 years) and mean body mass index was 29.9 kg/m^2^ (range, 19.4-47.9 kg/m^2^). Of the patients, 58% were women. All cases were performed for advanced osteoarthritis.

### Surgical technique

A medial parapatellar approach was used for all cases. Following removal of anterior and posterior cruciate ligaments, medial and lateral menisci, and all accessible osteophytes, navigation trackers were attached to the femur and tibia with pins, and anatomic landmarks for the femur and tibia were registered using the robotic system (Robotic Orthopedic Surgical Assistant, ROSA; Zimmer-Biomet, Warsaw, IN) using an “imageless” protocol that did not require preoperative imaging, in accordance with manufacturer guidelines [8]. The robotic system was utilized with an anterior-referencing workflow and instruments for all the cases in this study.

### Assessment method #1

**Figure 1:**
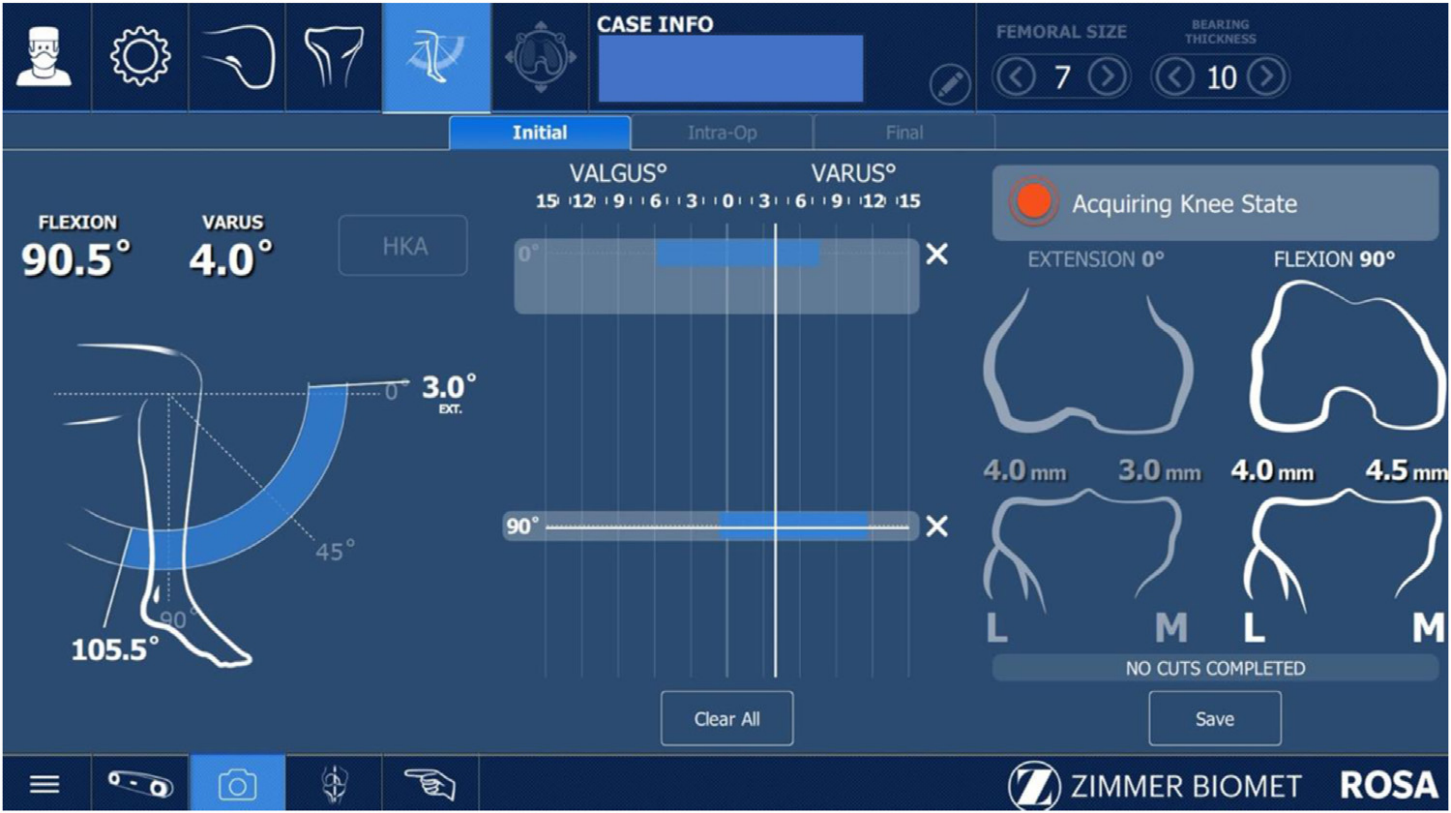
Live assessment of flexion gap laxity during the SURGEON technique.

**Figure 2:**
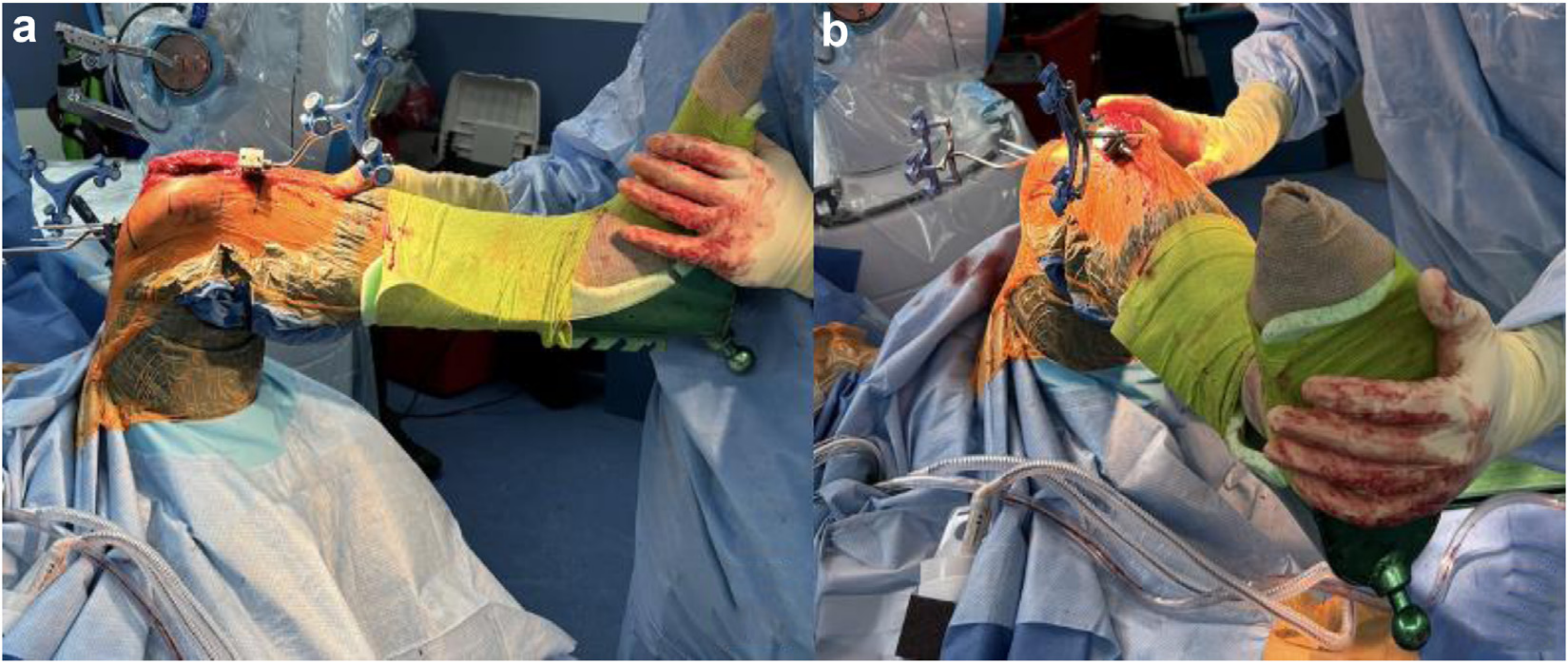
SURGEON technique demonstrating (a) internal rotation of the hip to assess maximum laxity in the medial compartment and (b) external rotation of the hip to assess maximum laxity in the lateral compartment.

Once bony landmarks were registered, a manual surgeon-applied stress laxity test (SURGEON) was performed by applying varus to valgus forces first in extension, then between 90 and 95° of flexion, and the maximal medial and lateral compartment laxity (quantified to the nearest 0.5 mm by the robotic software) was recorded (Fig. 1). To acquire the maximal laxity of the medial compartment in flexion, the hip was internally rotated until it locked, allowing distraction across the medial compartment without further rotational torque (Fig. 2a). Similarly, the maximal laxity of the lateral compartment in flexion was recorded with external rotation of the hip until it locked and then further distraction across the lateral compartment (Fig. 2b).

**Figure 3:**
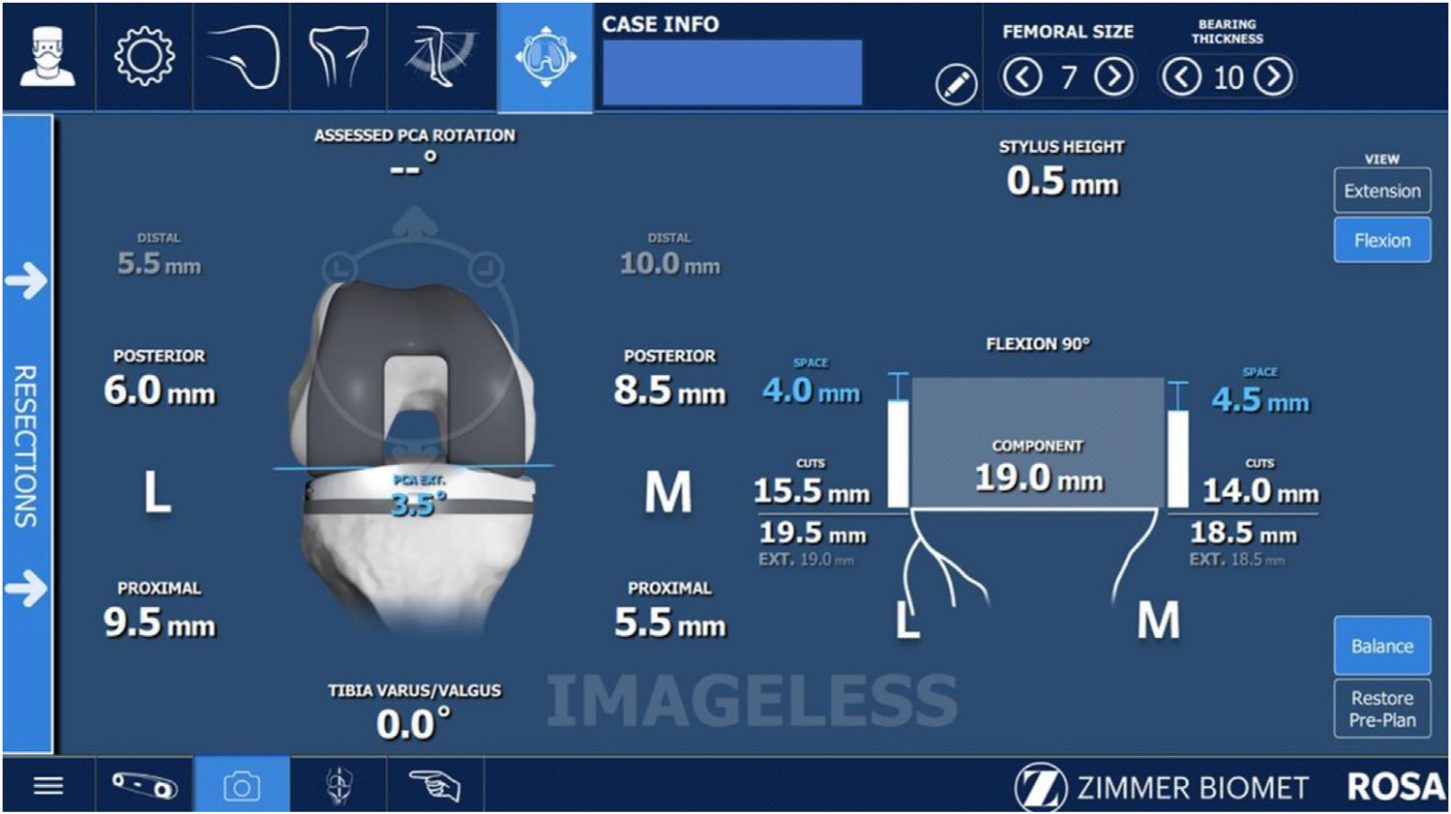
ROSA planning screen after the SURGEON flexion gap laxity technique.

Data acquired from this initial bony and soft tissue registration were used to plan bone coronal and sagittal alignment, resections, and choose appropriate size, rotation, and positioning of the femoral and tibial components, with the goal of balanced gaps in both flexion and extension within 1 mm (Fig. 3). Planned femoral size, femoral component rotation relative to the posterior condylar axis (PCA) (estimated to the nearest 0.5 degrees), and the AP position of the femoral component relative to the anterior cortex (estimated to the nearest 0.5 mm) were recorded.

### Assessment method #2

**Figure 4:**
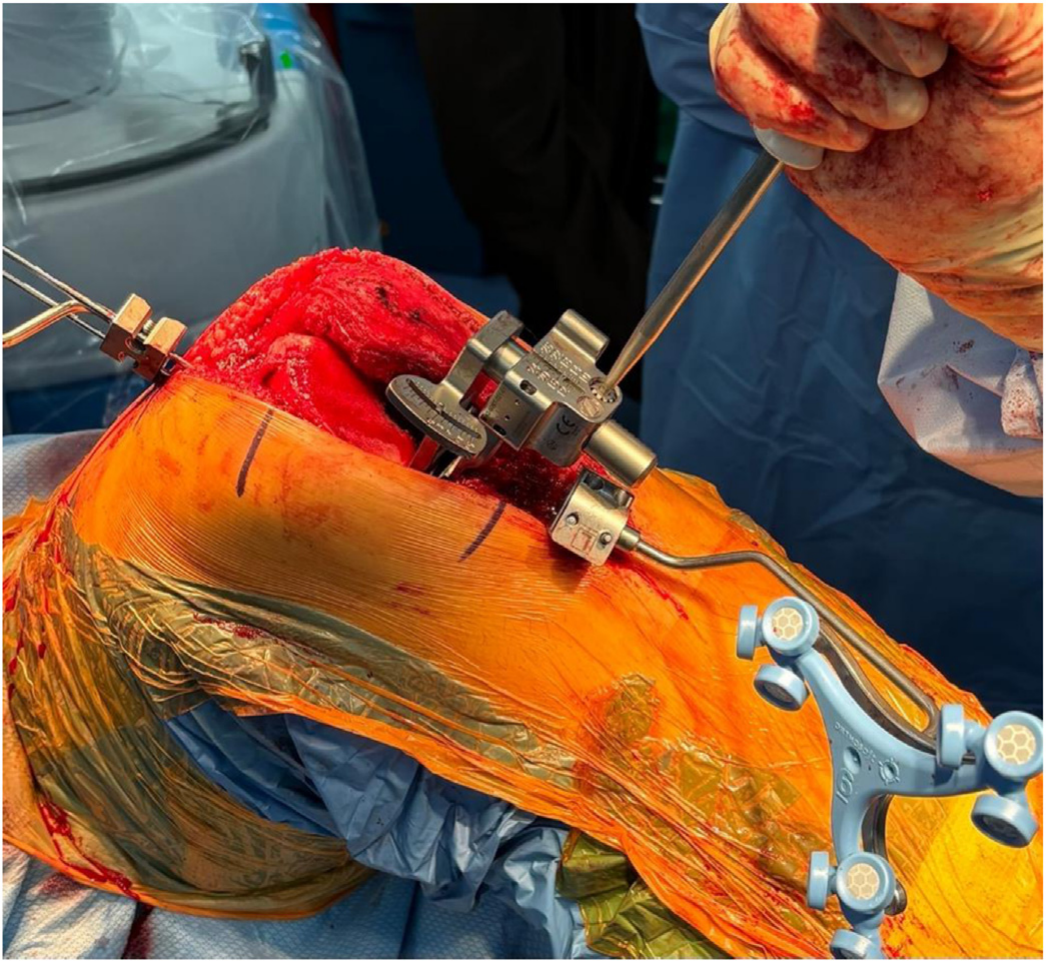
TENSOR technique demonstrating use of the ligament tensor tool.

**Figure 5:**
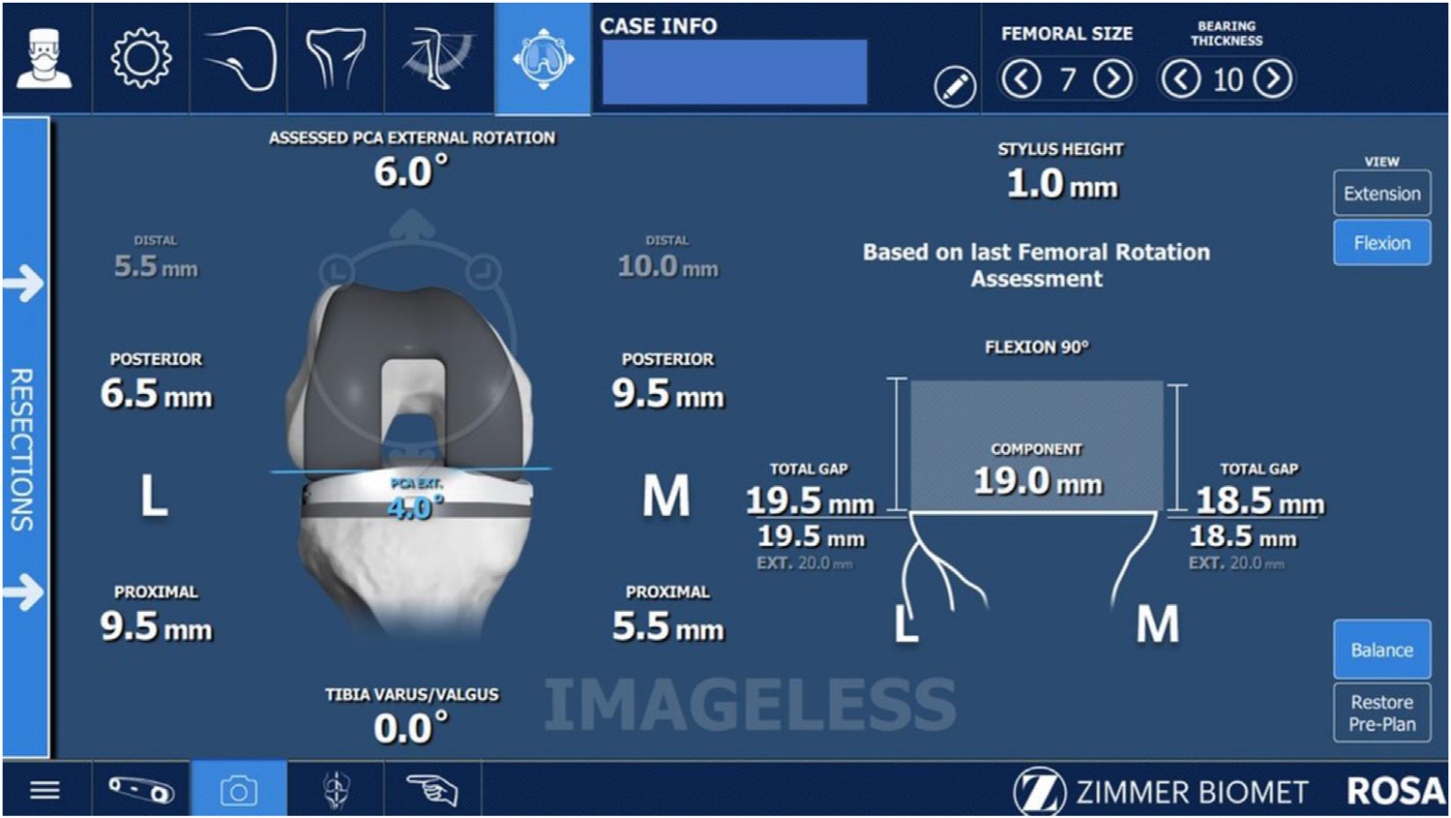
ROSA planning screen after the TENSOR flexion gap laxity technique.

Distal femoral and proximal tibial resections were then made with robotic assistance and validated. Following preparation and confirmation of a balanced extension gap, the flexion gap was assessed a second time using a different assessment technique incorporating a ligament tensor instrument (FuZion; Zimmer-Biomet; Warsaw, IN) prior to committing to the flexion gap cuts (TENSOR). A tensor instrument was chosen in the current study because it is the technique that, anecdotally, is most commonly employed by surgeons and is the technique that is recommended by the robotic manufacturer in training manuals and videos [8,9]. The instrument was placed between the prepared proximal tibia and the posterior femoral condyles with the knee positioned between 90 and 95° of flexion, and used to apply a consistent force to the flexion space (Fig. 4). This gap balancing instrument is designed to distribute a single force equally across medial and lateral compartments to assess laxity in both compartments simultaneously. The robotic software recorded this flexion laxity assessment in both compartments, and using the robotic software, the flexion gap was then re-planned for size, rotation, and positioning with the same goal of balanced gaps (Fig. 5). These new flexion gap data of planned femoral size, femoral component rotation, and AP position of the femur were recorded as well.

The flexion gap was then prepared using the femoral 4-in-1 guide positioned robotically. If there was a difference in recommended plan between the SURGEON and TENSOR methods, one was chosen based on the surgeon’s discretion and then pinned using the robotic arm. In addition to the component positioning variables for each method of flexion laxity assessment, the operative time to perform each technique (in seconds) was recorded.

### Statistical analysis

Paired-samples *t*-tests and a simple linear regression were conducted at a 95% confidence interval with a significance value of *P* < .05. SPSS software version 28.0.0.1.0 (Chicago, IL) was used for statistical analyses.

## Results

Paired-samples *t*-tests revealed no statistically significant differences in femoral component sizing between the SURGEON and TENSOR methods, with a mean difference of 0.06 sizes *(P* = .569). The knee system used offered femoral components that increased in 2 mm increments in the AP dimension with increasing size. There were 47 patients (94%) with an identical recommendation for femoral size, while 3 patients had a maximum planned deviation of one size between methods (Table 1).

**Table 1:** Paired-samples *t*-tests of measured variables between surgical techniques.

	SURGEON mean (range)	TENSOR mean (range)	*P*-value	Deviation mean (range)
Femoral size	Size 9	Size 9	.569	0.06 sizes ( ± 1 size)
External rotation (degrees)	4.0 (1.5-7.0)	4.0 (0.5-6.5)	.741	1.03 (−3.0 to +3.0)
Anterior flange (mm)	0.5 (0-1.0)	0.5 (0-1.0)	.785	0.13 (−0.5 to +0.5)
Operating room time (seconds)	20 (11-30)	40.0 (20-61)	<.001	21 (+4 to 38)

**Figure 6:**
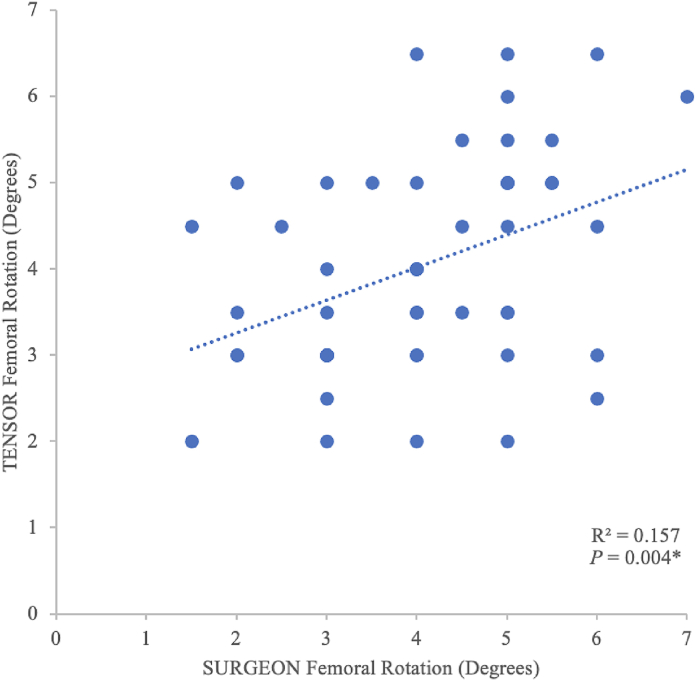
Femoral component rotation between surgical techniques.

Regarding femoral component rotation, paired-samples *t*-tests revealed no statistically significant differences between the SURGEON vs TENSOR methods (*P* = .741). Both methods produced identical means of 4.0 degrees external relative to the posterior condylar axis. The SURGEON vs TENSOR methods produced external rotation that ranged from 1.5 to 7.0 degrees and 2.0-6.5 degrees, respectively, relative to the posterior condylar axis. Planned femoral component rotation was identical in 13 patients (26%) and varied in 37 patients (74%). The mean variation between the SURGEON and TENSOR methods within the same patient was 1.0 degrees, and the maximum deviation ranged from −3.0 to +3.0 degrees (Table 1). SURGEON femoral component rotation could predict TENSOR femoral component rotation (R^2^ = 0.157; 95% confidence interval = 0.124, 0.633; *P* = .004) (Table 2, Fig. 6).

**Table 2:** Simple linear regression model of femoral component rotation between surgical techniques.

Unstandardized β (SE)	95% CI	*P-*value	R^2^
0.378 (0.127)	0.124, 0.633	.004	0.157

CI, confidence interval.

Regarding A-P femoral positioning, paired-samples *t*-tests revealed no statistically significant differences between the SURGEON vs TENSOR methods (*P* = .785). The mean anterior flange measurement for both methods was also identical at +0.5 mm proud of the anterior cortex. Both the SURGEON and TENSOR methods produced an anterior flange measurement that ranged from +0 to +1.0 mm with respect to the anterior cortex. Planned femoral component A-P position within the same patient was identical in 37 patients (74%) but deviated a maximum of −0.5 to +0.5 mm between the 2 methods (Table 1).

Regarding the operative time it took to perform each of the flexion gap assessments, the TENSOR method took significantly longer operating room time (mean = 40 seconds, range = 20-61 seconds) than the SURGEON method (mean = 20 seconds, range = 11-30 seconds, *P* < .001 (Table 1).

## Discussion

The current study found that using 2 different techniques for assessing flexion soft tissue laxity during RA-TKA produced near-identical results for planning flexion gap resections (eg, femoral component size, femoral component rotation, and A-P positioning). This has been a very limited topic of investigation in the literature to date. One recently published study by Maciag et al. compared femoral component rotation achieved with a TENSOR method applied during conventional TKA to that achieved with a SURGEON method applied during RA-TKA, but these 2 assessment techniques were applied in 2 *different groups of patients*. [10] In this study, the authors found a statistically significant difference of 1.6 degrees in femoral component rotation between groups [10], but it is unclear if there would have been differences if both techniques had been applied to the same patients. To the best of our knowledge, this is the only study published to date on this topic, and no other studies have compared different methods of assessing flexion gap laxity within the *same* patient.

Achieving accurate implant alignment and balanced gaps during TKA is important for optimizing function and overall quality of life [[11], [12], [13]]. An unbalanced flexion gap can lead to clinical repercussions such as effusions, pain, weakness, and instability during flexion-related activities and has one of the lowest improvement rates after revision TKA compared to other indications for revision TKA [14]. Specifically related to the flexion gap, femoral component rotation is an important variable that can affect implant survival and clinical outcomes, such as varus-valgus stability, patellar tracking, and patellofemoral contact forces [10,[15], [16], [17], [18], [19], [20]]. A gap-balancing philosophy, which was used in both the SURGEON and TENSOR arms in the current study, has the goal of positioning implants so that there is relative symmetry between the medial and lateral gaps in extension and flexion, and is a reliable and well-studied method. Using a gap-balancing technique to plan femoral component rotation has shown to increase component stability, function, and survivorship [[21], [22], [23]]. Similarly, balanced flexion gaps, compared to unbalanced gaps, have been shown to improve postoperative ROM and faster recovery improvement rates, even when the unbalanced cohort had a better ROM preoperatively [24]. While femoral component rotation affects flexion gap balance and has been well studied with respect to outcomes, the size and AP position of the femoral component affect how loose or tight the flexion gap will be relative to the extension gap and are also of importance during gap balancing.

When comparing the medial and lateral flexion laxity, RA-TKA has been shown to improve the rate of symmetric compartment balance and decrease the rate of overstuffed compartments [25]. Following bony resection, differences in medial and lateral flexion gaps were reported to be within 2 mm of each other in 99% of patients within a cohort of 335 patients when using RA-TKA. [5] Additionally, the same study has shown that intraoperative robotic assistance can accurately predict femoral component size within one size in 98% of patients [5]. Improving intraoperative planning accuracy is crucial for patient satisfaction, so early studies that suggest a higher level of improvement in quality of life (QoL) measures and Knee Society Scores associated with RA-TKAs may not be surprising [[26], [27], [28]]. Given these encouraging early results about gap balancing with RA-TKA, it is important to investigate some of the nuances surrounding different methods to assess soft tissue gaps in RA-TKA to see how variation between different surgical techniques might lead to different results.

The SURGEON technique, performed as a dynamic, surgeon-applied stress assessment, is commonly utilized by many surgeons who perform RA-TKA. In a previously published study, this technique was shown to be reliable for gap assessment both internally within the same surgeon across repeated measurements as well as across different surgeons at different levels of training, with an interclass correlation coefficient of 0.994 for interrater reliability [29]. The SURGEON technique is subjective to surgeon effort, trying to achieve maximum distraction through manual force short of rupturing ligaments. The previous study utilized the same SURGEON assessment technique; the only significant inter- or intra-observer variability came during bony registration, not the amount of stress applied by each surgeon, suggesting the SURGEON technique is very reproducible for assessment of flexion gap laxity [29].

The current study is the first known to compare this commonly utilized SURGEON technique in a multi-variate nature against the TENSOR technique, another well-studied and reliable gap assessment method. Tensor instruments have been shown to accurately assess laxity gaps and produce reliable outcomes in RA-TKA. The predicted preresection gaps vs the actual postresection gaps when using the tensor instrument during robotic-assisted TKA have not been shown to be significantly different in either the medial or lateral compartment [7]. Furthermore, tensor instruments have also been shown to be reliable for planning femoral component rotation. One well-designed study demonstrated that using tensor instruments results in relatively consistent external rotation of the femoral component for symmetrical compartments in flexion, at 4.83 ± 3.29 degrees relative to the PCA for all knees, 4.38 ± 3.23 degrees for varus knees, and 6.0 ± 3.21 degrees for valgus knees [29]. External rotation of the femoral component with respect to the PCA has been well studied to produce improved varus-valgus stability, patellar tracking, patellofemoral contact, and component survival [[16], [17], [18], [19], [20]]. These outcomes have been seen more reliably using the tensor tool compared to other techniques, such as size-guided measured resection and spacer block techniques, which cause relative internal rotation. Internal rotation of the femur with respect to the PCA has been correlated with symptomatic flexion instability in patients who seek revision TKA [30], as well as patellofemoral complications such as lateral tracking, patellar tilting, patellar subluxation, and dislocation/prosthesis failure [19,31]. The findings of the current study revealed how the SURGEON method accurately predicted the femoral component rotation of the TENSOR method within the same patient, with a mean of 4.0 degrees of external rotation for both surgical techniques.

The current study is the first known study to compare the SURGEON and TENSOR techniques of flexion gap assessment during RA-TKA in the same patient. There has been an increase in the number of femoral component sizes available with modern implants, with several systems offering 2 mm incremental sizing, as studies have shown that proper component sizing and position in the anteroposterior plane are important for the implant outcomes and longevity [32]. Intraoperative decisions about femoral component sizing and AP position occur before femoral 4-in-1 resections, and imprecise decisions have clear clinical implications, regardless of whether anterior or posterior referencing systems are used for femoral resections. When using anterior referencing systems, under-sizing can lead to a looser flexion gap and cause flexion instability, while oversizing can lead to tighter flexion gaps and limit ROM [32,33]. When using posterior referencing systems, under-sizing can lead to anterior notching and increase the risk of periprosthetic fracture, while oversizing can lead to overstuffing of the patellofemoral joint, which can limit ROM [32,[34], [35], [36]]. Furthermore, while RA-TKA has demonstrated increased accuracy in assessing sizing and positioning, the SURGEON and TENSOR methods during RA-TKA in the current study show that both techniques produce near-identical recommendations for femoral size and AP reference points.

Regarding operative time, the SURGEON method was significantly faster by about 20 seconds when compared to the TENSOR method. While saving operative time is both economically and procedurally efficient for the operating room and overall hospital, [37,38] and better for patient safety as decreasing surgical time has been shown to reduce the risk of infection [39], the clinical and economic significance of this small-time difference is not meaningful. Furthermore, while the gap assessment itself was shorter with RA-TKA, total operative time has shown to increase and become a limitation when utilizing RA-TKA [40]. Given the many benefits demonstrated with the use of robotics, efforts to decrease operative time in RA-TKA deserve further study.

The current study is robust in its prospective nature and internal controls of the same patient and the same surgeon. The results further strengthen the accuracy and reliability of gap assessment using the SURGEON technique in RA-TKA [10,29]. Additionally, it was the first known study to compare multiple variables that affect the flexion gap including component size, AP position, and component rotation between 2 commonly used preresection flexion gap soft tissue assessment techniques. Furthermore, these results strengthen the body of literature regarding RA-TKA by comparing a manual stress assessment that is widely utilized but has not been well studied (the SURGEON method) to previously well-studied techniques (the TENSOR method) [10,41].

The single-surgeon study design, while a strength for internal validity, was also a limitation, and seeing similarly consistent results across multiple surgeons would strengthen these conclusions. Furthermore, there were only 2 methods compared in the current study, and they were not compared against other well-studied techniques for gap laxity assessment, such as manual distraction, the use of a laminar spreader, or other instrument distraction methods such as using an elevator, osteotome, or “spoons.” In addition, the TENSOR technique was performed after the proximal tibia and distal femur cuts, which may have provided a slightly different laxity assessment than the SURGEON method, which was performed before these cuts were made. The impact of retained osteophytes and additional soft tissue releases after distal femoral and tibial bone cuts on flexion gap laxity is unknown. Although we do not expect this would have changed our outcomes. While the paired-samples *t*-test showed no statistically significant difference between the SURGEON and TENSOR methods, the patient-specific maximum deviations reveal there were still small (but not significant) differences. Lastly, potential confounding effects of knee alignment / morphology were not examined in the current study. The authors believe the internal control of the same knee across both techniques minimizes the concern for these effects, but this could be a good direction for future studies.

## Conclusions

Manual surgeon-applied stress assessment of flexion laxity compared with a ligament tensor produced near-identical flexion laxity data in RA-TKA, suggesting surgeons may comfortably choose either method as a reliable method of assessing soft tissue laxity of the flexion space to aid in planning the implant position. Future studies can continue to expand on the research on various assessment techniques and how improvements in assessing flexion laxity in RA-TKA can be made.

## Conflicts of interest

N. O. Sarpong is a paid consultant for Link Orthopaedics. R. P. Shah is a paid consultant for DePuy, Link Orthopaedics, Monogram, and Zimmer; has stock options in Parvizi Surgical Innovations; and is a board/committee member of the American Association of Hip and Knee Surgeons and the U.S. Food and Drug Administration. P. K. Sculco is a speaker bureau of DePuy, A Johnson & Johnson Company, EOS Imaging, and Intellijoint Surgical; is a paid consultant for DePuy, A Johnson & Johnson Company, EOS Imaging, Intellijoint Surgical, Lima Corporate, and Zimmer; has stock options in Intellijoint Surgical and Parvizi Surgical Innovation; and receives research support from Intellijoint Surgical. H. J. Cooper is a 3M speaker; is a paid consultant for DePuy, 3M, Zimmer-Biomet, Canary, and Polaris; has stock options in Polaris; receives research support from Smith & Nephew; is an editorial board member of the Journal of Bone and Joint Surgery (American); and is a board/committee member of the American Academy of Orthopaedic Surgeons and Eastern Orthopaedic Association. All other authors declare no potential conflicts of interest.

For full disclosure statements refer to https://doi.org/10.1016/j.artd.2024.101450.

## CRediT authorship contribution statement

**Catelyn A. Woelfle:** Writing – review & editing, Writing – original draft, Methodology, Investigation, Formal analysis, Data curation, Conceptualization. **Travis R. Weiner:** Methodology, Investigation, Conceptualization. **Peter K. Sculco:** Writing – review & editing, Investigation, Conceptualization. **Nana O. Sarpong:** Writing – review & editing, Methodology, Investigation. **Roshan P. Shah:** Writing – review & editing, Methodology, Investigation. **H. John Cooper:** Writing – review & editing, Project administration, Methodology, Investigation, Data curation, Conceptualization.

## Appendix A Supplementary Data

Conflict of Interest Statement for Woelfle

Conflict of Interest Statement for Cooper

Conflict of Interest Statement for Sarpong

Conflict of Interest Statement for Sculco

Conflict of Interest Statement for Shah

Conflict of Interest Statement for Weiner
