# Supplementary material for: Surgeon-Applied Stress and a Ligament Tensor Instrument Provide a Similar Assessment of Preresection Flexion Laxity During Robotic Total Knee Arthroplasty
Source: Arthroplast Today. 2024 Jul 3;28:101450. doi: 10.1016/j.artd.2024.101450 (PMC11283011; doi:10.1016/j.artd.2024.101450)
Supplement: Conflict of Interest Statement for Cooper [file mmc2.docx]

# INDIVIDUAL CONFLICT OF INTEREST STATEMENT

***American Association of Hip and Knee Surgeons***

(Adopted from the American Academy of Orthopaedic Surgeons disclosure statement)

The following form **must be filled out completely and submitted by each author (example, 6 authors, 6 forms).**

**All items require a response. If there is no relevant disclosure for a given item, enter "*None*.”**

**Manuscript Title: Surgeon-Applied Stress and a Ligament Tensor Instrument Provide a Similar Assessment of Pre-Resection Flexion Laxity During Robotic TKA**

1. Royalties from a company or supplier (The following conflicts were disclosed)

(none)

2. Speakers bureau/paid presentations for a company or supplier (The following conflicts were disclosed)

3M

3A. Paid employee for a company or supplier (The following conflicts were disclosed)

(none)

3B. Paid consultant for a company or supplier (The following conflicts were disclosed)

DePuy, 3M, Zimmer-Biomet, Canary, Polaris

3C. Unpaid consultants for a company or supplier (The following conflicts were disclosed)

(none)

4. Stock or stock options in a company or supplier (The following conflicts were disclosed)

Polaris

5. Research support from a company or supplier as a Principal Investigator (The following conflicts were disclosed)

Smith & Nephew

6. Other financial or material support from a company or supplier (The following conflicts were disclosed)

(none)

7. Royalties, financial or material support from publishers (The following conflicts were disclosed)

(none)

8. Medical/Orthopaedic publications editorial/governing board (The following conflicts were disclosed)

Journal of Bone and Joint Surgery (Am)

9. Board member/committee appointments for a society (The following conflicts were disclosed)

American Academy of Orthopaedic Surgeons, Eastern Orthopaedic Association

**Each author must sign AND print or type his/her name, date and submit a separate form**


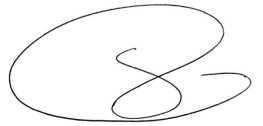
In addition, one BLINDED Conflict of Interest form (no author names used) should be submitted per manuscript with all author disclosures.

H. John Cooper 02/07/24

Author Name (Print or Type) Author Signature Date
