# Supplementary material for: Surgeon-Applied Stress and a Ligament Tensor Instrument Provide a Similar Assessment of Preresection Flexion Laxity During Robotic Total Knee Arthroplasty
Source: Arthroplast Today. 2024 Jul 3;28:101450. doi: 10.1016/j.artd.2024.101450 (PMC11283011; doi:10.1016/j.artd.2024.101450)
Supplement: Conflict of Interest Statement for Sarpong [file mmc3.docx]

# INDIVIDUAL CONFLICT OF INTEREST STATEMENT

***American Association of Hip and Knee Surgeons***

(Adopted from the American Academy of Orthopaedic Surgeons disclosure statement)

The following form **must be filled out completely and submitted by each author (example, 6 authors, 6 forms).**

**All items require a response. If there is no relevant disclosure for a given item, enter "*None*.”**

**Manuscript Title: Surgeon-Applied Stress and a Ligament Tensor Instrument Provide a Similar Assessment of Pre-Resection Flexion Laxity During Robotic TKA**

1. Royalties from a company or supplier (The following conflicts were disclosed)

(none)

2. Speakers bureau/paid presentations for a company or supplier (The following conflicts were disclosed)

(none)

3A. Paid employee for a company or supplier (The following conflicts were disclosed)

(none)

3B. Paid consultant for a company or supplier (The following conflicts were disclosed)

Link Orthopaedics

3C. Unpaid consultants for a company or supplier (The following conflicts were disclosed)

(none)

4. Stock or stock options in a company or supplier (The following conflicts were disclosed)

(none)

5. Research support from a company or supplier as a Principal Investigator (The following conflicts were disclosed)

(none)

6. Other financial or material support from a company or supplier (The following conflicts were disclosed)

(none)

7. Royalties, financial or material support from publishers (The following conflicts were disclosed)

(none)

8. Medical/Orthopaedic publications editorial/governing board (The following conflicts were disclosed)

(none)

9. Board member/committee appointments for a society (The following conflicts were disclosed)

(none)

**Each author must sign AND print or type his/her name, date and submit a separate form**

In addition, one BLINDED Conflict of Interest form (no author names used) should be submitted per manuscript with all author disclosures.


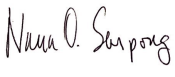


Nana O. Sarpong 02/07/24

Author Name (Print or Type) Author Signature Date
